# DEAD-Box RNA Helicases in Cell Cycle Control and Clinical Therapy

**DOI:** 10.3390/cells10061540

**Published:** 2021-06-18

**Authors:** Lu Zhang, Xiaogang Li

**Affiliations:** 1 Department of Nephrology, Renmin Hospital of Wuhan University, Wuhan 430060, China; zhanglu.1@foxmail.com; 2 Department of Internal Medicine, Mayo Clinic, 200 1st Street, SW, Rochester, MN 55905, USA; 3 Department of Biochemistry and Molecular Biology, Mayo Clinic, 200 1st Street, SW, Rochester, MN 55905, USA

**Keywords:** DEAD-box RNA helicases, cell cycle, treatment, DDX5, DDX3

## Abstract

Cell cycle is regulated through numerous signaling pathways that determine whether cells will proliferate, remain quiescent, arrest, or undergo apoptosis. Abnormal cell cycle regulation has been linked to many diseases. Thus, there is an urgent need to understand the diverse molecular mechanisms of how the cell cycle is controlled. RNA helicases constitute a large family of proteins with functions in all aspects of RNA metabolism, including unwinding or annealing of RNA molecules to regulate pre-mRNA, rRNA and miRNA processing, clamping protein complexes on RNA, or remodeling ribonucleoprotein complexes, to regulate gene expression. RNA helicases also regulate the activity of specific proteins through direct interaction. Abnormal expression of RNA helicases has been associated with different diseases, including cancer, neurological disorders, aging, and autosomal dominant polycystic kidney disease (ADPKD) via regulation of a diverse range of cellular processes such as cell proliferation, cell cycle arrest, and apoptosis. Recent studies showed that RNA helicases participate in the regulation of the cell cycle progression at each cell cycle phase, including G1-S transition, S phase, G2-M transition, mitosis, and cytokinesis. In this review, we discuss the essential roles and mechanisms of RNA helicases in the regulation of the cell cycle at different phases. For that, RNA helicases provide a rich source of targets for the development of therapeutic or prophylactic drugs. We also discuss the different targeting strategies against RNA helicases, the different types of compounds explored, the proposed inhibitory mechanisms of the compounds on specific RNA helicases, and the therapeutic potential of these compounds in the treatment of various disorders.

## 1. Introduction

### 1.1. Cell Cycle

**Figure 1:**
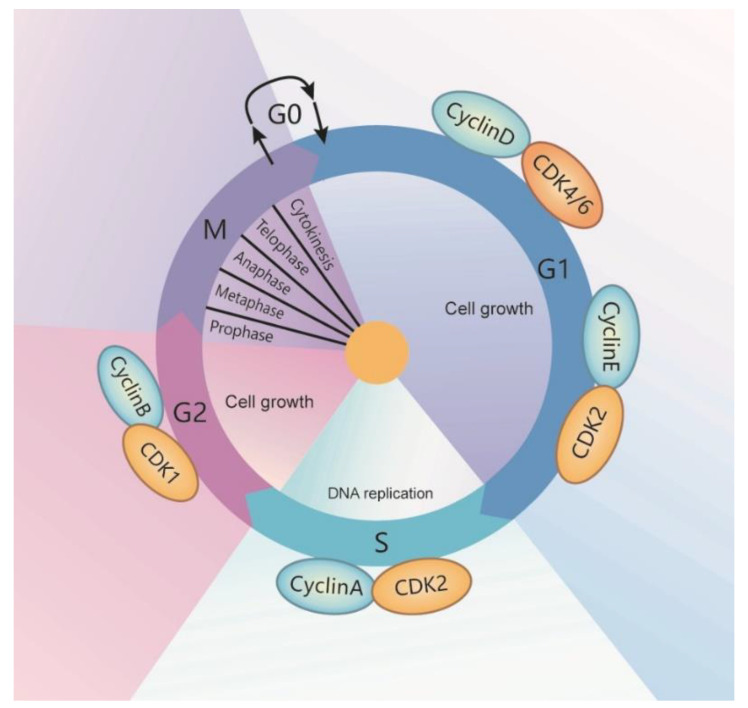
Schematic representation of the cell cycle. The cell cycle is divided into interphase (G1, S, G2) and mitotic phases. Nondividing cells are in G0. Cell cycle progression is controlled by cyclin/cyclin-dependent kinase (CDK) complexes in specific cell cycle phases.

The cell cycle is a series of events that occur in the interphase and mitotic phase (M-phase) [1,2]. The interphase is the longest phase of the cell cycle in which the genetic material gets duplicated and the cell prepares for division. The interphase includes three sub-phases: (1) G1-phase; the first-gap phase. In the G1-phase, cells grow in size, synthesize cell organelles and other proteins, and accumulate sufficient energy to prepare for cell division. (2) S-phase; the synthesis and DNA-replication phase. The centrosome and DNA are duplicated and give rise to the mitotic spindle during this phase. (3) G2-phase; the second gap phase. In the G2 phase, cells accumulate energy and grow further in size. Following the interphase is a period called the mitotic phase (M-phase). The M-phase includes two different critical processes: mitosis and cytokinesis. Mitosis consists of four sub-phases: prophase, metaphase, anaphase, and telophase. The cell divides the nucleus and a complete set of chromosomes into two daughter cells during mitosis. The cytoplasm splits and forms two independent cells in the process of cytokinesis [3,4] (Figure 1). The cell cycle is regulated by a series of cell cycle regulatory proteins. Cyclins and cyclin-dependent kinases (CDKs) families are key regulators of cell cycle progression [5]. At least nine CDKs are identified in animal cells, in which CDK1, CDK2, CDK3, and CDK4 are involved in the direct regulation of the cell cycle [6]. In general, CDKs that are active at each stage partner with cyclins to regulate cell cycle progression. CDK1 drives the cell cycle of G2 and M-phase with its partners, cyclin A2 and cyclin B1. CDK2 drives the cell cycle of G1, S, and G2 phases with its partners, cyclin A, cyclin D, and cyclin E. CDK3 drives the cell cycle of G0 phase with its partner, cyclin C. CDK4 and CDK6 drive the cell cycle of G1 phase, with its partners, cyclin D (D1, D2, and D3) and cyclin E (Figure 1). 

During the cell cycle, there are three checkpoints that occur at the end of G1 and the transition of G2/M, and in metaphase. The G1 checkpoint blocks the entry of the cell cycle into the S phase if a cell does not fulfill all the conditions, which lets the cell try to solve the conditions, or lets the cell enter into the G0 phase. The G_2_ checkpoint bars the entry of the cell cycle into the mitotic phase if certain conditions are not met, which ensures that all of the genetic material has been replicated and is not damaged. A damaged DNA will halt the cell cycle to let the damaged DNA be repaired. The M checkpoint, also named as the spindle checkpoint, is a point close to the end of the metaphase stage to make sure whether each pair of sister chromatids is correctly anchored to the two spindle microtubules—if not, the cycle will be halted. Recent studies suggest that RNA helicases are involved in these processes.

### 1.2. RNA Helicases

**Figure 2:**
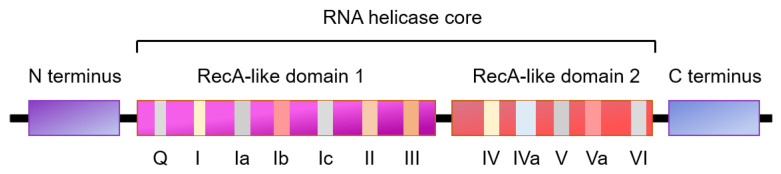
The sequence motifs of DEAD-box RNA helicases family are conserved. The DEAD-box RNA helicases family is characterized by a minimum of 12 conserved domains to form the DEAD-box helicase core, which consists of two RecA-related domains. Domain I and II contains 7 and 5 sequence motifs, respectively. The motifs of Q, I, II, and VI function as ATP binding and hydrolysis, and the motifs of Ia, Ib, Ic, IV, IVa, and V function as RNA binding. The motifs III and Va function as coordination between RNA and ATP binding. Domain II includes the DEAD motif (asp-Glu-ala-asp). The DEAD-box RNA helicases usually contain N and C terminal extensions, which determine their interaction with specific RNA and/or protein.

RNA helicases are ubiquitous, highly conserved enzymes that involve in nearly all aspects of RNA metabolism. RNA helicases use ATP to bind or remodel RNA, RNA secondary structure or ribonucleoprotein (RNP) complexes that are associated with RNA transcription, degradation, translation initiation, mRNA splicing, and ribosome biogenesis [7,8,9]. RNA helicases are grouped into various superfamilies dependent on the contents of conserved motifs. Most of RNA helicases relate to the superfamily 2 (SF2) of helicases comprising eleven subfamilies, five of which are termed the DExH/D helicases (DEAD-box, RIG-I-like DExH, SKI2-like DExH, viral DExH, and DEAH/RHA) with a conserved motif (Asp-Glu-Ala-Asp/His) [10]. Members of the DEAD-box (DDX) and DExH box families share a similar three-dimensional core structure containing a minimum of 12 conserved amino acid motifs, comprised of two tandemly repeated RecA-like domains [7,9]. The RecA domain 1 includes the ATP binding motifs (Q motif, I motif, and II motif), the ATP hydrolysis motif III, and the RNA-binding motifs (Ia motif and Ib motif) (Figure 2). The RecA domain 2 consists of the RNA-binding motifs (IV and V) and motif VI, which may regulate ATPase and unwinding activities [9,11]. Previous studies indicate that motifs Ia and Ib are structurally similar to motifs IV and V, respectively, which should have similar functions [12]. The *N*- and/or *C*-terminal extensions of most of the RNA helicases are able to interact with specific RNA or protein cofactors. This family of DEAD-box RNA helicases is able to unwind and restructure RNA molecules in an ATPase-dependent manner (Figure 2) [13,14]. 

RNA helicases play important roles in diverse biological processes. First, RNA helicases can regulate gene expression through catalyzing RNP complex rearrangements at the initiation of gene transcription and then are involved in gene post-transcriptional expression via regulation of ribosome biogenesis, RNA export, translation initiation and termination, and mRNA degradation [13]. Most of the RNA helicases move along a single strand of RNA to unwind secondary structures and displace other bound RNAs or proteins along the way. However, some DEAD-box RNA helicases, such as eIF4A (eukaryotic initiation factor 4A) are able to bind directly to the RNA double-strand and melt it [13,15]. Second, RNA helicases can also regulate cap-dependent translation initiation of mRNAs with a complex 5′UTR structure [16]. For example, eIF4A is required for the translation of oncogenes that contain complex secondary structures in their 5′ untranslated region through unwinding the 5′UTR (e.g., MYC, NOTCH1, MYB, CDK6, MDM2) [16,17,18]. In eukaryotes, the eIF4F translation initiation complex consists of three subunits: eIF4E, eIF4A, and eIF4G, which recruits the 40S ribosomal unit to the 5′ m7G-cap structure of the mRNA. Third, the DEAD-box containing RNA helicases, including DDX1, DDX5, DDX17, DDX20, DDX21, and RHA, can function as either transcriptional co-activators or co-repressors via interaction with spliceosome components [8,13,19].

The DEAD-box containing RNA helicases also play important roles in the pathophysiology of viral infection, aging, autosomal dominant polycystic kidney disease (ADPKD), neurological disorders, and cancer. First, the reduction of DDX3 is positively linked with hepatitis virus infection, especially HBV [20]. UAP56 or its paralog URH49 prevents the accumulation of dsRNA during influenza A virus infection via unwinding RNA [21]. Second, RNA helicase DDX5 (also known as p68) is usually downregulated in the aged mouse brain, indicating an association of p68 function with aging [22]. Third, we recently showed that p68 is upregulated in *Pkd1* mutant renal epithelial cells and tissues to regulate cystic renal epithelial cell proliferation and renal fibrosis through activation of the PKD-associated pathways, including ERK, mTOR, Rb, and TGF-β1 [19]. Forth, studies have confirmed that mutation of DDX3 is a significant cause of neurodevelopmental issues, including intellectual disabilities, hypotonia, autism spectrum disorder (ASD), corpus callosum malformations, microcephaly, and seizures [23]. In addition, RNA helicase DDX6 promotes neuronal differentiation by increasing the activity of the microRNA Let-7a [24]. Last, DDX5 has been implicated in several tumors (e.g., NSCLC, breast cancer, gastric cancer, multiple myeloma, glioma) [25,26,27,28,29] through aberrant expression or through the regulation of proliferation, metastasis, and invasion pathways that directly regulate oncogenesis. The precise transition between cell cycle phases is important for development, and the dysregulation of cell cycle results in diverse human diseases, such as promoting oncogenesis [30]. In this review, we will highlight the main functions of RNA helicases in the regulation of cell cycle, and discuss the inhibitors of RNA helicases, including eIF4A, DDX3 and DDX5, as promising therapeutic strategy through regulation of cell cycle [31,32,33,34].

## 2. The Role of RNA Helicases in Regulation of Cell Cycle Progression

### 2.1. RNA Helicases Regulate G1-S Phase Transition

In the cell cycle, the G1 phase is the first gap during which the cell prepares for DNA replication. In the G1 phase, the activity of CDKs promotes DNA replication and initiates G1/S phase transition. In general, cyclin D/CDK4/6 complexes play a critical role in the G1 phase; cyclin E/CDK2 complex plays a critical role in the G1 to S transition [35]. In the early G1 phase, growth factor signaling promotes the synthesis of cyclin D, then induces the formation of cyclin D/CDK4/6 complexes, resulting in the activation of CDKs [36]. Cyclin D/CDK4 and cyclin E/CDK2 complexes sequentially phosphorylate retinoblastoma protein (RB) (inactive form), leading to the release E2F from Rb-E2F complex and the activation of E2F [37]. The activated E2F then regulates the transcription of genes involved in cell cycle progression, including CDK2, cyclins A and E, and DNA synthesis, to promote G1/S transition [38,39]. During DNA damage, p53 acts as a transcription factor to upregulate the expression of p21, leading to cell cycle arrest in the G1/S phase [40]. Different RNA helicases are involved in transcriptional, translational, and post-translational regulation of the expression of cell cycle regulators.

**Figure 3:**
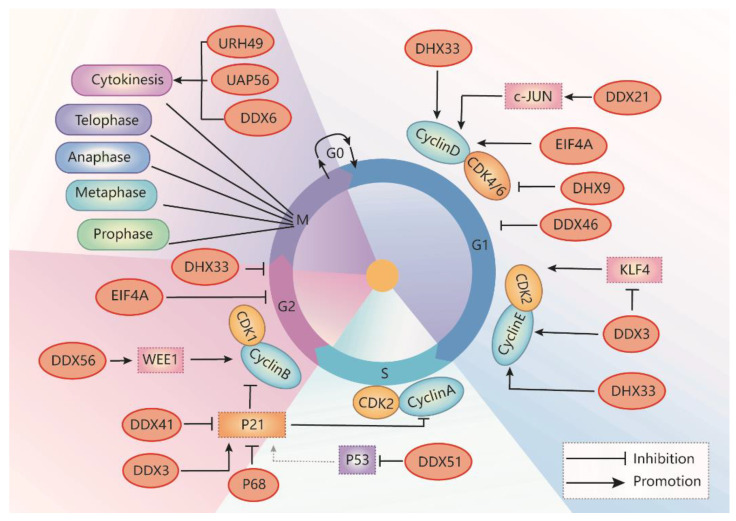
Schematic representation of the RNA helicases which are involved in the regulation of the cell cycle. RNA helicases are involved in G1/S transition: DDX3 positively regulates cyclin E1 translation but negatively regulates KLF4 expression to increase CDK2 expression. DHX33 initiates the transcription of E2F1, cyclin E2, cyclin D1, MMP9, MCMs, CDC6, and CDC20. DHX9 decreases the transcription of CDK6, leading to CIZ1 nuclear translocation. DDX21 activates c-Jun transcription, resulted in the increase of the synthesis of cyclin D1 mRNA. eIF4A regulates G1/S transition through regulation of the translation of cyclin D1, cyclin D2, and CDK6. RNA helicases are involved in S phase progression: DDX51 promotes S phase progression, possibly through negative regulation of cell-cycle-related proteins, p53-p21. RNA helicases are involved in G2-M phase transition: DDX56 promotes G2/M transition via the increase of intron retention and tumor suppressor WEE1 expression. RNA helicases are involved in mitotic phase progression: Knockdown of UAP56 or URH49 leads to mitosis defect. DDX6/CGH-1 functions to regulate microtubule cytoskeleton and chromosome separation. RNA helicases regulate the expression of CDK inhibitor p21: DDX41 and p68 inhibit p21 transcription and translation, respectively.

DDX3/Ded1 is one of the most widely studied DEAD-box RNA helicases. DDX3 regulates RNA metabolism, including transcription, pre-mRNA splicing, RNA export, and translation, which plays a critical role in many biological processes [20]. DDX3 also promotes phase separation when it interacts with ATP [41]. DDX3 also regulates cell apoptosis via the p53 signaling pathway during embryonic development in animal models [42]. Dysregulation of DDX3 plays a critical role in various diseases, including inflammation, viral infection, neurological disorders, and cancer [41,43]. The role of DDX3 in oncogenesis is related to the regulation of cell growth, cell cycle, and cell survival. Increased DDX3 expression promotes cancer cell growth in medulloblastoma, colorectal, breast, prostate, and lung cancer [20,44,45,46], whereas depletion of DDX3 induces cell cycle arrest in the G1 phase in cancer cells of those cancers (Table 1) [20,31,47,48,49]. Knockdown of DDX3 also inhibits cell cycle progression by blocking entry into the S phase. Mechanically, DDX3 facilitates translation initiation of the cell cycle regulator cyclin E1 mRNA via its 5′ UTR [50]. In addition, DDX3 inhibits the expression of Krüppel-like factor 4 (Klf4) by altering its mRNA splicing, resulting in an upregulation of the expression of CCNA2 and CDK2 [31]. In sum, DDX3 promotes G1/S transition by promoting cyclin E1 translation, suppressing KLF4 expression, and promoting CDK2 expression (Figure 3).

DHX33 plays a significant role in ribosome RNA synthesis and mRNA translation. DHX33 promotes numerous cellular processes, including cell cycle progression, apoptosis, and migration, by promoting the transcription of a subset of genes, including Bcl2, MMP9, MMP14, and urokinase-type plasminogen activator [51,52]. DHX33 is required for promoting cell cycle progression at the G1-to-S phase transition, which is overexpressed in several types of human cancers, such as lung cancers, hepatocellular carcinoma, lymphoma, colon cancer, and glioblastoma (Table 1) [16,51,52,53,54]. A deficiency of DHX33 induced a significant increase of G1-phase cells and a marked decrease of S-phase cells in different cancer cells [51,53,54,55]. Mechanically, DHX33 recruits active RNA polymerase II (Pol II) to the promoters of many cell-cycle-related genes, including cyclin E2, cyclin D1, E2F1, MMP9, MCMs, CDC6, and CDC20, and initiates the transcription of those genes [53,55] (Figure 3). 

DHX9, as a multifunctional protein, regulates transcription, translation, miRNA processing, RNA transport, and genome maintenance processes [7,8,13,45]. DHX9 is overexpressed in several types of human cancers, including cervical cancer, breast cancer, prostate cancer, colorectal cancer, hepatocellular carcinoma, and Ewing sarcoma [56,57,58,59]. Knockdown of DHX9 arrests cell cycle at G0/G1phase (Table 1) [60]. DHX9 negatively regulates the expression of CDK6, an important protein for G1/S transition and cell division, through binding to the 3’-untranslated region (3’UTR) of CDK6 mRNA to decrease its stability and reducing polyribosome incorporation, resulting in the decrease of G1/S transition (Figure 3) [61]. In addition, DHX9 interacts with CIP1-interacting zinc finger protein 1 (CIZ1) to induce the translocation of CIZ1 to the nucleus during the S phase [62], which is necessary for the progression of the cell cycle [62]. DHX9 also regulates cell proliferation through interaction with EGF receptor, cAMP-response element-binding protein, BRCA1, and RNA polymerase II [60,63]. 

DDX21, as an important nucleolar protein, plays a critical role in ribosomal RNA biogenesis and transcriptional regulation [64]. DDX21 is a transcriptional activator of c-Jun via their direct interaction to regulate mRNA expression of the downstream target genes, including EGFR and cyclin D1 [65]. DDX21 is dysregulated in colon cancer, lymphomas, neuroblastoma, and some breast cancers (Table 1) [17,66,67,68]. Upregulation of DDX21 promotes breast cancer cell proliferation by activation of the transcription factor AP-1 to regulate the transcription of cyclin D1 and rRNA processing, resulting in the increase of cells in the S phase (Figure 3) [65,69]. Deletion of DDX21 arrests cell cycle at G1/S transition in Hela cells [69]. In addition, DDX21 interacts with PARP-1 and results in its ADPRylation by PARP-1. Inhibition of PARP decreases the activity of DDX21 and suppresses cell proliferation in breast cancer cells [70].

The eIF4A family proteins regulate RNA biology, including transcription, translation, and degradation of mRNA [71,72]. eIF4A is also important for gene expression through translational regulation. The ribosome profiling analysis has identified eIF4A regulated genes that act at the G1/S phase transition, including Cyclin D1, Cyclin D2, and CDK6 (Figure 3) [73]. There are three paralogous genes of eIF4A, including eIF4A1, 2, and 3. eIF4A3 functions as an RNA-binding protein (RBP) and is a core component of the exon junction complex (EJC), which is mainly localized in the nucleus (Table 1) [74,75]. eIF4A3 is highly expressed in colorectal cancer [76]. The binding of eIF4A3 to LncRNA H19 prevents the recruitment of eIF4A3 to the mRNAs of the cell cycle regulators, including cyclin D1 and cyclin E1, for post-transcriptional modification, resulting in an acceleration of colon cancer cell growth [73,76].

DDX46, also named Prp5, has multiple functions in nuclear pre-mRNA splicing through the hydrolysis of ATP to rearrange local RNA–RNA or protein–RNA interactions [77]. The expression of DDX46 is upregulated in colorectal carcinoma, esophageal squamous cell carcinoma, gastric cancer, and osteosarcoma cells, and knockdown of DDX46 inhibits cancer cell proliferation, invasion and induces cell apoptosis (Table 1) [78,79,80,81]. Silence of DDX46 arrests cell cycle at G1 phase in esophageal squamous cell carcinoma cell lines (Figure 3) [79]. In addition, knockdown of DDX46 significantly reduced the phosphorylation of Akt and IκBα. Whether DDX46 regulates G1/S phase transition through Akt and IκBα needs to be further investigated. 

### 2.2. RNA Helicases Regulate S Phase Progression

During the S phase of the cell cycle, genetic materials, such as DNA, are synthesized for the duplication of the chromatin and reproduction of the whole genome, which is a pre-requisition for cell division. Once cells enter the S phase, cyclin E/CDK2 complex needs to be silenced to eliminate the DNA re-replication [82]. Dissociation of CDK2 from cyclin E/CDK2 complex results in its association with the newly synthesized cyclin A to form CDK2/cyclin A complex, which can phosphorylate proteins that are necessary for completion of the S phase. Alternatively, spliced p53 isoform (Delta-p53) transactivates the expression of endogenous p21, resulting in the inhibition of CDK2/cyclin A activity to and attenuate S phase progression [83].

Thus far, only one RNA helicase, DDX51, has been associated with S phase progression. It has been reported that downregulation of DDX51 leads to cell cycle arrest in the S phase (Figure 3) [84], probably through DDX51 mediated rRNA process and other signaling pathways. During the rRNA process, DDX51 interacts with pre-60S complexes and promotes the removal of U8 snoRNA from pre-rRNA, which is required for ribosome maturation [85]. DDX51 is also a negative regulator of p53, and thereby actively promotes cell proliferation [86]. In addition, DDX51 can promote breast cancer cell proliferation by increasing the activity of the Wnt/β-catenin signaling pathway [26]. Moreover, DDX5 can promote cell proliferation by increasing the transcription of cyclin D1 in non-small cell lung carcinoma (Table 1) [87].

### 2.3. RNA Helicases Regulate G2/M Phase Transition

In the cell cycle, the G2 phase is the second gap for cells to prepare for mitosis (M phase). During the G2 phase, cyclin A is degraded, but cyclin B is synthesized, resulting in the association of Cdc2 with cyclin B, which is required for the initiation of mitosis. Cyclin B can be phosphorylated at Tyr15 by WEE1, a kinase of the WEE family, resulting in altering equilibria and affecting G2/M transition [88,89]. The initiation of G2 arrest, triggered by DNA damage or inappropriate replication, is important for DNA damage repair, which can be regulated through p53 mediated signaling pathways [90]. In addition, p21, as the downstream target of p53, is able to inhibit the activity of Cyclin A, Cyclin D, and other cell-cycle-related proteins, including cdc2 [40]. Different RNA helicases are associated with G2/M arrest.

DDX56 regulates diverse RNA metabolism, including transcription, translation, ribosome biogenesis, pre-mRNA splicing, and mRNA degradation. DDX56 is a component of free nucleoplasm 65S preribosomal particles, which may participate in the assembly of the 60S ribosomal subunit. DDX56 has also been reported to regulate ribosome assembly through its association with the Oct4 and Sox2 complex, which is necessary to maintain embryonic stem cells (ESCs) proliferation [91]. DDX56 is upregulated in osteosarcoma and colorectal cancer (Table 1) [92,93,94]. Depletion of the DDX56 arrest cell cycle in the G2/M phase via the decrease of tumor suppressor WEE1 expression, which functions as a G2/M DNA damage checkpoint (Figure 3) [94], results in the inhibition of cell proliferation and clone formation and the induction of p53 mediated apoptosis in osteosarcoma cells [92,93]. However, whether DDX56 regulates the cell cycle through p53-p21 signaling needs to be further investigated.

Furthermore, some RNA helicases that we mentioned above are also involved in G2/M phase transition. It has been reported that knockdown of eIF4A3 decreases cell cycle arrest in the G2/M phase, but increases apoptosis [95]. DHX33 also positively regulates G2/M transition to promote cell cycle progression (Figure 3) [53].

### 2.4. RNA Helicases Regulate Cytokinesis

Cytokinesis is the last step of the cell cycle in which the cell must devotedly split the chromosomes and cytoplasm to produce two daughter cells with equal contents [96]. RNA helicases are involved in the key steps of cytokinesis, including the assembly and contraction of the contractile network, the formation of the mitotic spindle, and the interaction between the microtubule and cortical actomyosin cytoskeleton [97]. 

UAP56 and its paralog URH49 are 90% identical to each other. These RNA helicases are nuclear RNA helicases essential for mRNA export and splicing and piRNA biogenesis. They also localize at cytoplasm (Table 1). Knockdown of UAP56 often results in premature sister chromatid separation, whereas knockdown of URH49 leads to chromosome arm resolution defects and failure of cytokinesis (Figure 3) [98,99]. Knockdown of UAP56 affects the expression of distinct mRNAs necessary for normal mitosis, such as BRCA1, and knockdown of URH49 resulted in the downregulation of survivin/BIRC5 and PRC1 mRNAs to regulates cytokinesis [98]. Recently, it has been reported that URH49 also regulates cytokinesis by the regulation of the expression of two CPC complex components, AURKB and BIRC5, which are associated with the cell cycle [99]. 

DDX6, also named CGH-1 in *Caenorhabditis elegans* [100], is highly conserved from unicellular eukaryotes to vertebrates [100]. DDX6 functions as an oncogene in colorectal cancer and hepatocellular carcinoma (Table 1). Knockdown of DDX6 inhibits tumor cell growth through down-regulation of c-Myc [101]. CGH-1 and its orthologs are found in dynamic ribonucleoprotein complexes, which contributes to global and transcript-specific mRNA storage, translational repression during development and cell differentiation. CGH-1 is also localized to RNA-containing P-granules, which are RNA/protein condensates in the germline of *C. elegans* [102]. CGH-1 regulates the recruitment of Aurora B kinase and ZEN-4, between the separating chromosomes. Partial depletion of CGH-1 in worms results in assembly defects of interzonal microtubule bundles and the failure of cytokinesis that becomes remarkable after anaphase onset (Figure 3) [102]. 

### 2.5. RNA Helicases Target CDK Inhibitor p21

CDK inhibitors (CKIs) are mainly identified in CIP/KIP and INK4 families, which are key regulators of G1, S, and G2 phase transitions during the cell cycle [103]. The CKI inhibitors in the CIP/KIP family, including p21, p27, and p57, target CDK1, CDK2, CDK4, and CDK6. The CKI inhibitors in the INK4 family, including p15, p16, p18, and p19, also targets CDK4 and CDK6. The expression and stability of CKIs are regulated by multiple mechanisms, including transcriptional, post-transcriptional, epigenetic regulation, and ubiquitin-dependent or independent protein degradation. P21 is one of the most studied CDK1 inhibitors, which was first identified as a CDK-interacting protein (CIP1) and wild-type p53-activated factor 1 (WAF1). P21 can bind to cyclin A/CDK2 [104], cyclin E/CDK2 [105], cyclin D/CDK4 [106], and cyclin B/CDK1 complexes [107], resulting in cell cycle arrest in the S phase, G1/S transition, and G2/M transition. We will discuss how p21 is regulated by different RNA helicases.

DDX41 is involved in all aspects of RNA biology [108]. Mutation of DDX41 alters pre-mRNA splicing and RNA processing, resulting in the loss of its tumor suppressor function [109]. DDX41 can function as a repressor of p21, which can bind to the 3′UTR of the p21 mRNA to inhibit its translation of under basal and stress conditions (Figure 3). Mutation of DDX41 (G521S) results in the loss of its ATP hydrolysis activity and its ability to regulate p21 translation [103]. Mutation of DDX41 also induces alternative splicing and misexpression of cell cycle genes, such as cyclin E2, CKI 1A in erythroid progenitor cells, resulting in diminished proliferation and defective differentiation, finally leading to anemia and acute myeloid leukemia (Table 1) [109,110]. In addition, the DDX41 mutant can activate DNA damage response, leading to an Ataxia-telangiectasia mutated [17] and Rad3-related (ATR)-triggered cell cycle arrest [103]. These findings make DDX41 an important player in the regulation of the cell cycle and DNA damage response. 

RNA helicase DDX5/p68 is involved in multiple cellular processes, including transcription, pre-mRNA and rRNA processing, and miRNA processing. Overexpression of p68 promotes the development of breast cancer, colorectal tumors, prostate cancer, and leukemia, suggesting that p68 plays an important role in cancer development and progression (Table 1) [34]. DDX5/p68 can function as a transcriptional co-activator and can be recruited to the promoters of its target genes together with the activated transcription factors. In particular, p68 is selectively required for p53-dependent p21 expression by promoting the recruitment of p53 and RNA polymerase II to the p21 promoter, leading to cell-cycle arrest after DNA damage [111]. Knockdown of p68 enhanced the transcription of p21 but decreased the expression of the beta-catenin-regulated genes, including c-Myc, cyclin D1, and c-Jun [112]. Deletion of p68 results in a loss of the G1/S checkpoint, allowing cell cycle progression through the S to G2/M phases (Figure 3). We recently showed that p68, also via its interaction with p53, negatively regulates the expression of the Pkd1 gene to promote cystic cell proliferation and inhibit apoptosis in ADPKD [19,113]. Whether p68 is involved in the regulation of other cell-cycle-related factors in ADPKD needs to be further investigated. In addition, DDX5 also interacts with Tat, an RNA binding protein, and may enhance the availability of P-TEFb through expropriating HEXIM1, and acting as a necessary cellular factor for efficient HIV transcription elongation [114]. 

DDX3 has also been identified as a positive regulator of p21 [41,42]. Overexpression of DDX3 decreases the S-phase population via upregulation of p21 transcription (Figure 3), resulting in the inhibition of lung cancer cell growth [42]. In contrast, downregulation or loss of nuclear localization of DDX3 promoted tumor growth in hepatocellular carcinoma [41]. DDX3 transactivates p21 promoter through the regulatory elements located upstream of the transcription start site, including six Sp1 sites, two E2F sites, and one AP-2 site [41]. DDX3 mediated transcriptional modulation on the p21 promoter depends on its ATPase activity but not helicase activity [41]. Thus, DDX3 inhibitors can affect the cell cycle through the regulation of the translation of cell cycle regulators, including cyclin E1 and KLF4, and key CDK inhibitor, p21. 

## 3. Development of RNA Helicase Inhibitors for Clinical Treatment

### 3.1. Different Targeting Strategies Are Used to Develop Compounds against RNA Helicases

The important roles of RNA helicases in the regulation of the cell cycle, cell proliferation, cellular transformation, apoptosis, and cell adhesion and motility make RNA helicases an active targets of drug development for the treatment of viral infections, neurodegenerative diseases, and cancers. Based on the crystal structures and the mechanism(s) of activity of the enzymes, specific and selective inhibitors for RNA helicases have been designed. In general, inhibitors targeting RNA helicase activity could act via one or more of the mechanisms: (1) inhibition of the ATPase activity of RNA helicases by interference with ATP binding and result in limiting the energy necessary for the unwinding and translocation; (2) inhibition of the helicase activity by competitively occupying the RNA binding site of the RNA helicases; and (3) stabilization of RNA helicases onto RNA, resulting in the inhibition of translation initiation.

#### 3.1.1. Compounds That Inhibit the ATPase Activity of RNA Helicase

RX-5902 is a first-in-class anticancer compound targeting phosphorylated p68 (Table 2). RX-5902 interacts with the Y593 of the phosphorylated p68, resulting in a large diminishment of nuclear β-catenin, and this regulation is dependent on the ATPase activity of p68 [115]. 

Compound **18** is an eIF4A inhibitor, which has been identified from a high throughput screening (HTS) campaign. Compound **18** selectively acts against eIF4A through competitive inhibition of its ATPase activity (Table 2) [116].

#### 3.1.2. Compounds That Inhibit the Helicase Activity of RNA Helicases

RK-33 (diimidazo[4,5-d:4′,5′-f]-[1,3]diazepine), a small molecule, is a ring-expanded nucleoside (REN) analogue. RK-33 is designed to selectively inhibit DDX3 activity but no other DEAD-box RNA helicases by binding to the ATP pocket of DDX3 (Table 2) [117]. Treatment with RK-33 reduces tumor growth or tumor cell proliferation in xenograft mouse models of Ewing sarcoma, lung cancer, and prostate cancer by inducing G1 cell cycle arrest and apoptosis [33,47,118]. NZ51 is another DDX3 inhibitor, which has been shown to competitively occupy the RNA binding site of DDX3 to suppress its RNA helicase activity without affecting its ATPase activity (Table 2). 

Hippuristanol, as a polyoxygenated steroid, selectively inhibits eIF4A through binding to its C-terminal domain [119]. Hippuristanol suppresses the RNA-binding activity of eIF4A by locking eIF4A in a closed conformation, but does not prevent its ATP-binding activity (Table 2) [72,120].

#### 3.1.3. Compounds That Stabilize RNA Helicases onto RNA, Resulting in the Inhibition of Translation Initiation

Both Silvestrol and CR-1-31-B have been identified as specific inhibitors of the eIF4A, which act as the part of the translation initiation complex that binds to the 5′-cap structure (Table 2) [32,73]. The potential mechanism for these compounds to inhibit eIF4A is through increasing the affinity of eIF4A to its target mRNA to stall eIF4A on its RNA substrate, which results in a consumption of eIF4A from eIF4F–mRNA complexes [32,73].

#### 3.1.4. Compounds That Regulate Both ATPase and RNA Helicase Activities

Pateamine A (Pat A) is designed as a biologically active metabolite, which selectively stimulates the RNA binding activity of eIF4A, resulting in disruption of the eIF4F complex and leading to inhibition of cap-dependent translation. Pat A also increases the ATPase and helicase activities of eIF4A [121]. Pat A can disrupt the interaction of eIF4A-eIF4G and free eIF4A from the eIF4F complex. In addition, Pat A also promotes the formation of the eIF4A homodimer and the interaction of eIF4A-eIF4B (Table 2) [121,122].

### 3.2. The Therapeutic Potential of RNA Helicase Inhibitors in the Treatment of Various Disorders

As described above, different inhibitors of RNA helicases have been developed, which may potentially be used in disease treatment [47,123,124]. Here, we mainly focus on inhibitors that target DDX3, eIF4A, and DDX5 (p68) in the treatment of diseases.

The DDX3 inhibitor, NZ51, is able to block the ATP-dependent helicase activity of DDX3. NZ51 suppresses DDX3 activity at low micromole concentration and displays anti-proliferative activity by inhibiting cell replication at the G1 phase in aggressive breast cancer cells [43,49]. However, treatment with NZ51 did not significantly reduce tumor growth in breast cancer mouse model [49]. Doxorubicin, an antitumor drug, can inhibit the ATPase activity of DDX3, which shows anticancer activity on human oral squamous cell carcinoma cells. However, the cardiotoxicity is a limitation for doxorubicin in vivo [125]. The DDX3 inhibitors, RK-33, can bind with the ATP-binding domain of DDX3 to block its helicase activity. RK-33 can also disrupt the DDX3-β-catenin complex, resulting in an interference of Wnt signaling and regulating cell differentiation, cell proliferation, and G1/S transition [33]. The highest RK-33 sensitivity was observed in tumor cells. Treatment with RK-33 inhibited the growth of colorectal cancer cell lines and promoted cell death with IC50 values ranging from 2.5 to 8 μM [48]. RK-33 also inhibits the growth of other cancer cells in vitro, including lung cancer, medulloblastoma, and prostate cancer [44,46,47,48]. Importantly, RK-33 can reduce not only the toxicities associated with the treatment but also the dose of radiation to 5 Gy in the medulloblastoma treatment of a mouse xenograft model [44], suggesting that RK-33 can increase radio-sensitization. The safety and efficacy of this combined treatment in human diseases should be evaluated in future clinical trials. Treatment with RK-33 also changes the Ewing sarcoma cellular proteome, including the proteins involved in DNA replication, mRNA translation, and proteasome function [126]. Treatment with RK-33 inhibits the growth of human Ewing sarcoma xenografts, without overt toxicity [126]. In addition, RK-33 attenuates the assembly of stress granules (SGs), one type of mRNA-containing cytoplasmic aggregates, formed in response to global translation arrest or to various stress conditions, such as reactive oxygen species, heat, starvation, and viral infection. Thus, RK33 may be relevant for the treatment of stress granules-dependent pathologies, including viral infection, tumor resilience, and neurodegeneration [117]. The Advancement of RK-33 to clinical trials may represent a novel therapeutic strategy, especially for cancer treatment in the future (Table 2).

Hippuristanol, as a pan eIF4A inhibitor, has been reported to inhibit human T lymphotrophic virus type 1-infected T-cell line and adult T-cell leukemia cell proliferation by promoting cell cycle arrest at the G1 phase and suppressing the expression of cell cycle proteins and cyclin-dependent kinases, and inducing apoptosis by decreasing the expression levels of Bcl-x, baculoviral IAP repeat, containing 3 X-linked inhibitors of apoptosis (xIAP) and caspase 8 and FADD like apoptosis regulator [127,128]. Treatment with hippuristanol significantly inhibited the growth and invasion of primary effusion lymphoma cells compared with untreated mice [129]. Silvestrol, a selective eIF4A inhibitor, has been reported to inhibit eIF4A in MDA-MB-231 cells, resulting in the blocking of cell cycle progression at the G1/S phase transition, possibly through a decrease in the expression of cyclin D1, cyclin D2, and CDK6, and the induction of apoptosis through inhibition of the translation of Bcl2 [73]. CR-1-31-B, another eIF4A inhibitor, suppresses a panel of relevant key cell-cycle regulators, including cyclin D1, CDK2, and CDK4, in MCF-7 and T47D cells at a low dose, and suppresses cyclin E1, cyclin A2, and CDK6 in those cells at higher doses. CR-1–31-B can enhance G1 phase arrest induced by CDK4/6 inhibition [32]. Moreover, treatment with allosteric eutomer inhibitors (T-595) of eIF4A3 results in cell cycle arrest at the G2/M boundary and increases apoptosis [95]. Pateamine A also inhibits the activity of eIF4A. Des-methyl, des-amino pateamine A (DMDA-PatA) is a structurally simplified analogue of the marine natural product pateamine A [130]. Treatment with DMDA-PatA rapidly stops DNA synthesis in the S phase, which shows potent anticancer activity in MDA-MB-435 cells and LOX melanoma cell lines [130,131]. These studies suggest that targeting eIF4A might be a promising therapeutic strategy for cancer treatment (Table 2). 

DDX5 (p68) can be phosphorylated at the Y593 site by growth factors, including platelet-derived growth factor, to increase cellular proliferation, epithelial to mesenchymal transition (EMT), malignant transformation, cell migration, and oncogenesis through translocation of β-catenin to nuclear and activation of cyclin D1, c-JUN, and c-MYC [132]. RX-5902 is a small molecule inhibitor of phosphorylated p68 (p-p68), which can decrease the nuclear localization of β-catenin in murine triple-negative breast cancer models [124]. Treatment with RX-5902 decreases the expression of genes that are regulated by the β-catenin pathway, such as c-Myc, cyclin-D1, and c-Jun, in cancer cells, resulting in the inhibition of cancer cell proliferation [115,133]. RX-5902 has been assessed in a first-in-human phase I dose-escalation study (clinical trial identification: NCT02003092) (Table 2). This study suggests that RX-5902 is well tolerated with a favorable side effect profile [134]. The recommended phase 2 dose for RX-5902 is 250 mg, which can be administered daily for 5 on/2 days off with continuous dosing in patients with metastatic triple-negative breast cancer [134]. The preclinical research and clinical trial indicate that RX-5902 might be a promising therapeutic strategy for breast cancer; the role of RX-5902 on other diseases needs to be further investigated.

## 4. RNA Helicases and Phase-Separated Organelles

Recently, several studies have demonstrated that the DEAD-box RNA helicases involved in regulation of liquid–liquid phase separation (LLPS) play a crucial role in the formation of RNA-containing membrane-less organelles, including pericentriolar material (PCM), stress granules (SGs), P bodies, and P granules in *Caenorhabditis elegans* [135].

Pericentriolar material is defined as a matrix of proteins surrounding the two barrel-shaped centrioles during mitosis, which serves as a platform for protein complexes to regulate organelle trafficking, protein degradation, and spindle assembly. PCM are highly dynamic, spherical organelles without a membrane. Zwicker et al. [136] used the concept of phase separation and proposed a theoretical description of centrosomes as liquid droplets. Gle1 is a highly conserved molecule of RNA-dependent DEAD-box ATPase proteins, which is enriched at the centrosome and basal body. Gle1 can be assembled into the toroid-shaped pericentriolar material around the mother centriole. Reduction of Gle1 is correlated with decreased pericentrin localization at the centrosome and microtubule organization defects that may be involved in the regulation of cell cycle progression through spindle assembly checkpoint [137]. 

Stress granules are RNPs that assemble in response to environmental stresses such as oxidative stress, heat shock, or osmotic shock. One component of yeast stress granules is Ded1p, also named DDX3 in mammalian. Temperature-driven liquid phase condensation of Ded1p induces the sequestration of housekeeping mRNAs and promotes an expanded heat shock response in program results of the preferential expression of stress proteins at 39 °C [138]. Targeting the ATPase activity of DDX3 by RK-33 reduces the assembly of SG, only marginally affect the disassembly of SGs [117]. In contrast, Begovich et al. [139] found that decrease of the ATPase activity of Ded1 promotes SG formation in an in vitro assembled SGs (IVSGs) system. They also showed that ATP not only suppresses IVSG formation but also act as an energy source for disassembly and remodeling of IVSGs. These findings suggested that ATP may act as a biological hydro trope to prevent proteins from phase separating, indicating a nonenzymatic role for ATP in SG assembly. Given the findings of ATP-dependent RNA helicases on the regulation of SG dynamics, this field might get more attention in the future. 

P bodies are the large cytoplasmic granules, which are membrane-less cytoplasmic organelles that form via phase-separation once RNAs and nearby RBPs assemble into ribonuclear particle (RNP) granules. P bodies have all the key characters of LLPS, which are liquid-like, spherical, and dynamic, and can be dissolved by the alcohol 1,6-hexanediol. One of the key regulators that regulates P bodies’ assembly is the DEAD-box ATPase Dhh1 (DDX6 in humans). Dhh1, as an enhancer of decapping, is involved in the translational repression of mRNAs. Dhh1 is involved in phase separation and controls P bodies dynamics in vivo, which is mainly dependent on its RNA-stimulated ATPase activity. The ATPase activator Not1 dissolves Dhh1 droplets in vitro and inhibits P-body formation in vivo [140]. P bodies’ formation can also be promoted by Pat1 through enhancing the Dhh1-regulated LLPS [141]. Pat1 is a conserved multi-domain RNA binding protein, which may directly counteract the Not1-stimulated ATPase activation of Dhh1 to regulate P bodies formation. A recent study indicated that the helicase activity of DDX6 is not only essential for P-body assembly but is also required for exit from the pluripotent state and for hPSC differentiation [142]. Furthermore, depletion of DDX6 on mesodermal progenitor revealed that thousands of genes were aberrantly expressed as examined by RNA-sequence (1366 genes upregulated; 1040 genes downregulated). Moreover, the upregulated genes were associated with cell differentiation, downregulated genes were related to cell cycle progression. However, the direct connection between cell cycle progression and P bodies needs to be further investigated. 

P granules are RNA/protein condensates in the germline of *Caenorhabditis elegans.* P granules are membrane-less organelles that may assemble by intracellular phase separation. GLH-1, a germline putative RNA helicase, regulates the formation and disassembly of P granules coupling with distinct steps of its ATPase hydrolysis cycle [143]. PGL-1 and PGL-3 are the self-assembling P-granule proteins, contain RG-repeats sequences. PGL-1 is also required to localize FBF-2 to perinuclear P granules and for efficient binding of FBF-2 to its mRNA targets, resulted in preventing translation of meiotic mRNAs [144]. In addition, DEAD-box helicases LAF-1 and VBH-1 also contain RG-repeats sequences, indicating that LAF-1 and VBH-1 may transiently associate with P granules [145]. 

DDXs globally promote phase separation in their ATP- and RNA-bound state. ATP hydrolysis induces the release of RNA clients from a DDX, results in the disassembly of RNA-containing membrane-less organelles [135]. The condensates formed by 2NT-DDX4^YFP^, a recombinant protein, dissolve during mitosis and leads to increasing of noise in protein concentration. In contrast, droplets reform in most postmitotic cells and decrease the noise in protein concentration. These findings indicate that the RNA helicases play an important role in phase separation [146]. The potential of RNA helicases mediated phase separation in cell cycle control is a perspective direction for further investigation. 

## 5. Conclusions

RNA helicases are highly conserved enzymes important for RNA metabolism, which are involved in multiple steps of cell cycle regulation (Figure 3). RNA helicases are involved in cell cycle regulation at each phase with different mechanisms of action, including the regulation of (1) pre-mRNA transcription or splicing of some cell cycle regulators, (2) translation of cell cycle stage associated cyclins and CDKs, and (3) transcriptional and post-translational modification of the effectors, such as p21, that are involved in cell cycle progression. For example, DDX3 regulates the expression of cyclin A1, cyclin E1, and CDK2. DHX33 regulates the transcription of many cyclins and CDKs. DDX21 regulates the expression of cyclin D1. DDX56 regulates the expression of WEE1, a G2–M cell cycle checkpoint gene. DDX5 (p68) is involved in the expression of cyclin D1, which is also important for p53 activation. DHX9 regulates the formation of cyclin D–CDK complex. DDX46 regulates G1/S cell cycle arrest. DDX41 is a repressor of CDK inhibitors, such as p21. The effect of RNA helicases on cell cycle progression is cell-type dependent, and targeting RNA helicases with inhibitors should have a significant effect on the control of cell cycle and cell proliferation. Small molecule inhibitors of RNA helicases have already been developed based on the crystal structures and the mechanism(s) of action of these enzymes. These inhibitors have been validated in vitro and in vivo, which may be further tested in different diseases, including cancer and ADPKD, as a potential therapeutic strategy.

## Figures and Tables

**Table 1:** RNA Helicases in each Cell Cycle Stage and their Intracellular Localization.

Cell Cycle Stage	RNA Helicases	Intracellular Location	Expression
G1-S phase transition	Ded1/DDX3	Nuclear speckles and cytoplasm	Upregulation in medulloblastoma, colorectal, breast, prostate, and lung cancer
	DHX33	Nucleus and nucleoli	Upregulation in lung cancers, hepatocellular carcinoma, lymphoma, colon cancer, and glioblastoma
	DHX9	Nucleus	Upregulation in cervical cancer, breast cancer, prostate cancer, colorectal cancer, hepatocellular carcinoma, and Ewing sarcoma
	DDX21	Nucleus and cytoplasm	Dysregulation in colon cancer, lymphomas, neuroblastoma, and breast cancers
	eIF4A	Nucleus and cytoplasm	Dysregulation in pancreatic cancer, breast cancer, prostate cancer
	DDX46	Focal nuclear	Upregulated in colorectal carcinoma, esophageal squamous cell carcinoma, gastric cancer, and osteosarcoma cells
S phase progression	DDX51	Predominantly in nuclear	Dysregulation in NSCLC
G2-M phase transition	DDX56	Nucleolus	Upregulation of DDX56 in various cancer, including osteosarcoma, colorectal cancer, and relates to a poor prognosis
	DHX33	Nucleus and nucleoli	See above
Mitosis	UAP56	Nucleus and cytoplasm	Not clear
	URH49	Nucleus and cytoplasm	Not clear
Cytokinesis	UAP56	Nucleus and cytoplasm	Not clear
	URH49	Nucleus and cytoplasm	Not clear
	DDX6	Nucleus and cytoplasm	colorectal cancer and hepatocellular carcinoma
Regulate the expression of p21(WAF1/CIP1)	DDX41	Nucleus and cytoplasm	DDX41 mutant leads to anemia and acute myeloid leukemia. DDX41 increased in cervical cancer.
	DDX5	Mostly in nucleus, cytoplasmic levels of DDX5 increased in the G2/M phase	p68 increased in a range of cancers except for hepatocellular carcinoma
	DDX3	Predominantly in nuclear speckles and at low levels in cytoplasm	See above

**Table 2:** The Structures and Sites of Action of RNA Helicase Inhibitors in Diseases.

RNA Helicases	Inhibitors	Chemical Structure	Mechanisms of Action	Diseases	Model	Toxicity or Tissue-Specific
DDX3	RK-33	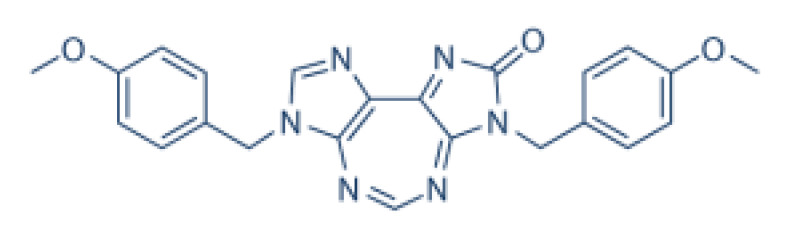	Inhibition of helicase activity	Lung cancer, medulloblastoma, prostate cancer, Ewing sarcoma, and colorectal cancer	In vitro, and animal models	No discernable toxicity in animal models
	NZ51	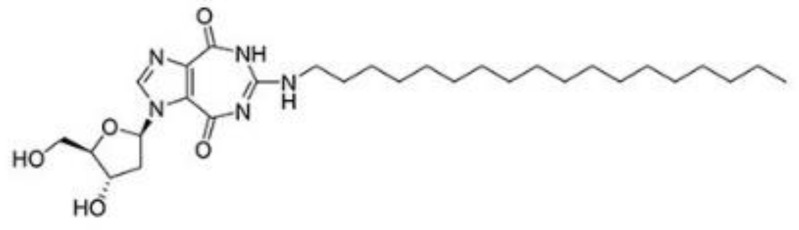	Inhibition of helicase activity	Breast cancer	In vitro	Not clear
	Doxorubicin	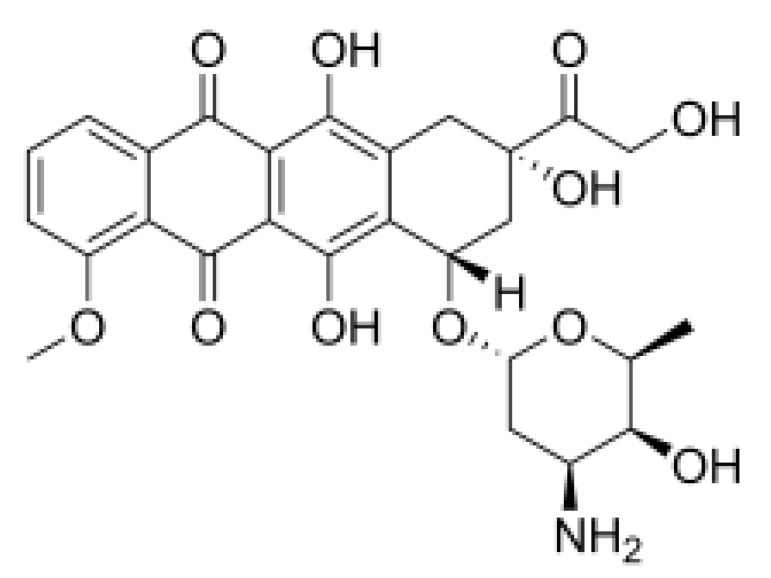	Inhibition of ATPase activity	Oral squamous cell carcinoma	In vitro	Cardiotoxicity
eIF4A	Compound 18	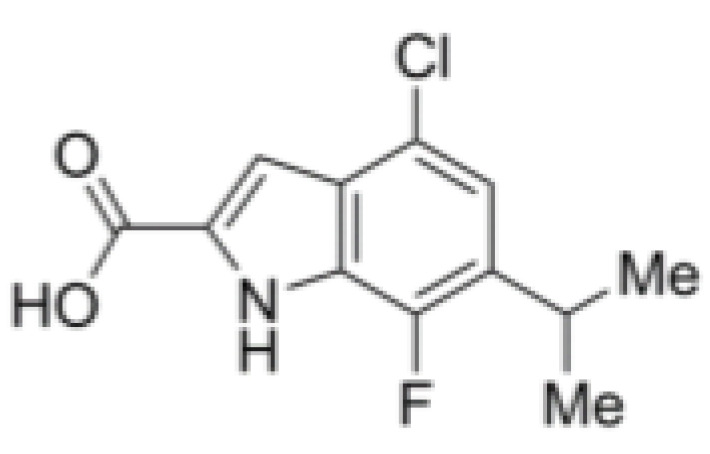	Inhibition of ATPase activity	Exon junction complex	NA	IC_50_: 0.97 μmol/L
	Silvestrol	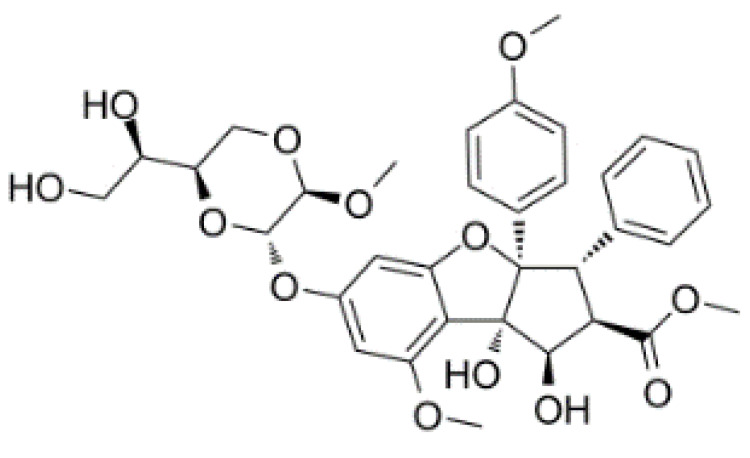	Stabilization of RNA helicase onto RNA	Breast cancer	In vitro	Not toxic in vitro and in vivo at concentrations of effective activity
	Hippuristanol	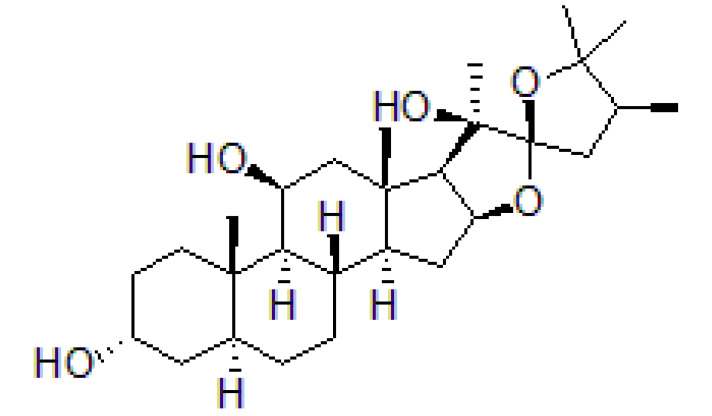	Inhibition of helicase activity	Leukemia	In vitro	Not clear
	CR-1-31-B	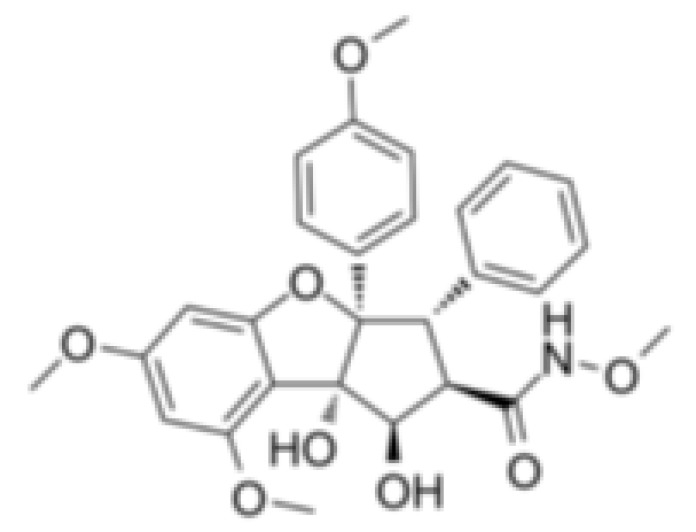	Stabilization of RNA helicase onto RNA	Breast cancer	In vitro	Not clear
	Pateamine A	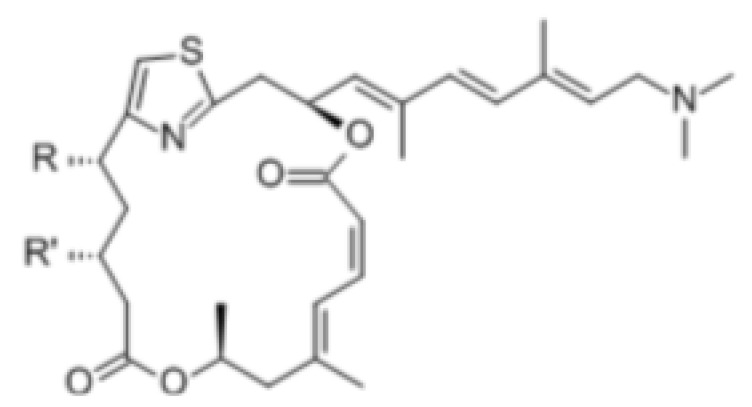	Regulation of ATPase and RNA helicase activity	Melanoma	In vitro, and animal models	Low toxicity to quiescent cells
DDX6	RX-5902	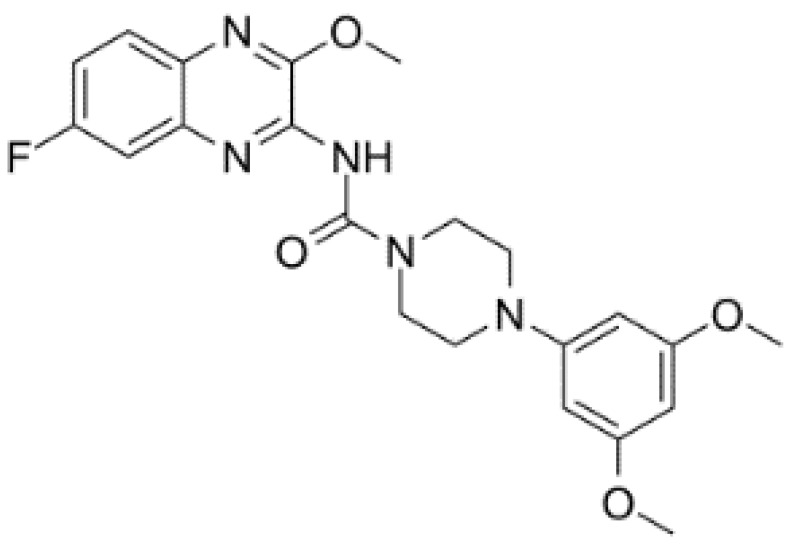	Inhibition of ATPase activity	TNBC	In vitro, preclinical models of TNBC, phase I study	Not clear

## Data Availability

Not applicable.
